# Re-biopsy by endobronchial ultrasound procedures for mutation analysis of non-small cell lung cancer after EGFR tyrosine kinase inhibitor treatment

**DOI:** 10.1186/s12890-016-0268-3

**Published:** 2016-07-26

**Authors:** Takehiro Izumo, Yuji Matsumoto, Christine Chavez, Takaaki Tsuchida

**Affiliations:** Department of Endoscopy, Respiratory Endoscopy Division, National Cancer Center Hospital, 5-1-1, Tsukiji Chou-ku, Tokyo, 104-0045 Japan

**Keywords:** EGFR-TKI, EBUS-TBNA, EBUS-GS, T790M, Lung cancer, Re-biopsy

## Abstract

**Background:**

Re-biopsy for resistant non-small cell lung cancer (NSCLC) after treatment with epidermal growth factor receptor-tyrosine kinase inhibitors (EGFR-TKIs) is important for selection of better therapy, but there have been no reports about the utility of endobronchial ultrasound (EBUS)-guided procedures for such purpose. The aim of this study was to evaluate the utility of EBUS-guided re-biopsy for resistant NSCLC after treatment with EGFR-TKIs.

**Methods:**

From January 2013 to December 2015, 53 consecutive patients who underwent EBUS-guided re-biopsy for mutation analysis of NSCLC after EGFR-TKI treatment were assessed.

**Results:**

Nine patients underwent EBUS-guided transbronchial needle aspiration (EBUS-TBNA) and 44 patients underwent EBUS with a guide sheath (EBUS-GS) transbronchial biopsy. The technical success rates were 100 %. As for mutation analysis, all 9 specimens (100 %) from EBUS-TBNA and 33 specimens (75.0 %) from EBUS-GS were adequate for gene profiling. The remaining 11 specimens from EBUS-GS procedures were inadequate for mutation analysis owing to the absence of tumor component in the sample (*n* = 6) or insufficient specimen (*n* = 5). There were no related severe complications.

**Conclusions:**

Re-biopsy by both EBUS-TBNA and EBUS-GS were useful and safe sampling procedures for mutation analysis of EGFR-TKI resistant NSCLC.

## Background

Epidermal growth factor receptor tyrosine kinase inhibitors (EGFR-TKIs) have been demonstrated to be effective in treating non-small cell lung cancer (NSCLC) patients with EGFR mutations [1–3]. EGFR-TKIs offer both good clinical response and survival benefit in NSCLC patients who have EGFR mutation [4]. However, a majority of the responders eventually develop acquired resistance to EGFR-TKIs [5].

Until now, several studies have uncovered several mechanisms of acquired resistance, such as secondary EGFR mutations (T790M mutation), mesenchymal-to-epithelial transition factor receptor (MET) amplification, and human epidermal growth factor receptor 2 (HER2) gene amplification [6]. Among these, a secondary missense T790M mutation was observed in nearly half of all cases that were resistant to EGFR-TKIs [7].

In November 2015, a third generation EGFR-TKI (Osimertinib; Astra Zeneca, London) has been approved by the US Food and Drug Administration (FDA) to treat patients with a type of advanced NSCLC that has a specific EGFR mutation, called T790M, and which has become worse after treatment with other EGFR-TKIs. Osimertinib has shown clinical effectiveness and tolerability in NSCLC patients with T790M mutation of EGFR [8].

Examining T790M is very important in treatment selection. Because EGFR-TKI treatment is indicated for advanced and unresectable NSCLC, examination for T790M mutation is usually performed on small biopsy specimens.

So far, most reports on re-biopsy procedures after EGFR-TKI resistance have been on computed tomography-guided transthoracic needle biopsy (CTNB), which is also the common first method of choice for the initial diagnosis of lung cancer [9]. However, complications, such as pneumothorax and bleeding, after CTNB have been reported [9].

Another biopsy approach is by bronchoscopy, but its use has been limited by a lower diagnostic accuracy compared with CTNB. Recently, endobronchial ultrasound (EBUS) guidance during bronchoscopy procedures, such as EBUS guided-transbronchial needle aspiration (EBUS-TBNA) and EBUS with a guide sheath (EBUS-GS) transbronchial biopsy, have been introduced and improved the diagnostic accuracy of bronchoscopy [10, 11]. However, there have been no reports about the utility of EBUS procedures for re-biopsy.

In this study, we evaluated the efficacy and safety of EBUS procedures for re-biopsy and mutation analysis of EGFR-TKI resistant NSCLC.

## Methods

### Subjects

This study was approved by the National Cancer Center Institutional Review Board. Written informed consent for the procedure was obtained from all patients. Consecutive patients who underwent re-biopsy by EBUS procedures for mutation analysis of NSCLC after EGFR-TKI treatment at the hospital between January 2013 and December 2015 were enrolled. There were 2907 patients in whom EBUS-guided bronchoscopy procedures were performed during the study period. Among these, 89 patients were for re-biopsy purposes. Among these re-biopsy patients, we selected NSCLC patients who were treated with at least one EGFR-TKI regimen. Disease progression during chemotherapy, based on the RECIST ver1.1 definition, was a criterion for inclusion; 36 patients were ineligible because of the absence of prior EGFR-TKI treatment.

### Methods and equipment

All bronchoscopies were performed via the oral route under local anesthesia with mild sedation by intravenous administration of midazolam.

EBUS-GS was performed for patients with peripheral pulmonary lesions (PPLs) without mediastinal and hilar lymphadenopathy; a GS kit (K-201 or K-203; Olympus Ltd, Tokyo, Japan) and a radial EBUS (R-EBUS) probe (UM-S20-20R or UM-S20-17S; Olympus Ltd, Tokyo, Japan) were used. The bronchial route was planned by reviewing the chest CT images before EBUS-GS. Virtual bronchoscopic navigation/simulation systems (Ziostation2, Ziosoft Ltd, Tokyo, Japan; LungPoint, Bronchus Ltd, Mountain View, CA, USA; or Bf-Navi, Olympus Ltd, Tokyo, Japan) were used to detect the bronchial route to the target PPLs. A PPL was defined as an abnormal growth surrounded by normal lung parenchyma and was bronchoscopically invisible. Upon reaching the target bronchus, the GS together with the R-EBUS probe was inserted through the working channel of the bronchovideoscope and was advanced towards the PPL under fluoroscopic guidance (VersiFlex VISTAVR; Hitachi Ltd, Tokyo, Japan). Ultrasound scanning was performed while manipulating the R-EBUS probe until the lesion was localized by the corresponding EBUS image. After confirming the location of the PPLs, the R-EBUS probe was removed while keeping the GS in place for the usual transbronchial sampling using forceps, brush, and/or transbronchial needle aspiration under fluoroscopic guidance. Lesion location was assigned based on a study of Baaklini et al. [12], but with some modifications. With the area around the hilum on CT as reference, lesions in the inner and middle third ellipses were designated as central parenchymal location, whereas lesions in the outer third ellipse were designated as peripheral parenchymal location. The bronchus sign on CT was defined as the presence of a bronchus leading directly to a PPL [13].

EBUS-TBNA was performed for patients who had mediastinal and hilar lymphadenopathy. EBUS–TBNA was performed using either one of the following 22G needles: Vizishot (NA-201SX-4022; Olympus Ltd, Tokyo, Japan) or SonoTip EBUS Pro with stainless steel (GUS-45-18-022; Medi-Globe Ltd, Germany). The convex probe EBUS (CP-EBUS; BF-UC260FW, Olympus Ltd, Tokyo, Japan) was inserted through the oral route in the same way as during usual bronchoscopy. The ultrasound images were generated using a dedicated ultrasound processor (EU-ME1 or EU-ME2; Olympus Ltd, Tokyo, Japan). When the target lesions were visualized by CP-EBUS, the TBNA needle was inserted through the working channel of the bronchoscope and was advanced to penetrate the tracheobronchial wall into the target lesion under real-time EBUS guidance. Aspiration was done by moving the needle back and forth inside the target lesion for 20–30 times, under negative pressure. After sampling, suction was released before retracting the needle from the scope.

### Pathologic evaluation and mutation analysis

Cytology and histology specimens were sent for pathologic examination. Positive diagnostic criteria suitable for molecular analysis were defined by the presence of malignant cells based on histologic features or based on class IV/V on cytology examination by Papanicolaou stain. Overall detection rate was based on a positive diagnosis by histology and/or cytology.

EGFR mutation analyses of the genomic DNA extracted from tumor samples were performed using the Scorpion ARMS method.

### Statistical analysis

Descriptive statistics were presented as frequency, percentage, and median (range). Univariate analyses were performed using Fisher’s exact test. Multivariate analysis using logistic regression was performed to determine the factors associated with the yield. Variables selected via univariate analyses (P value <0.10) were evaluated using multivariate logistic regression analysis. A two-tailed P value <0.05 was considered to indicate statistical significance. Correlation of study variables were performed with EZR (Saitama Medical Center, Jichi Medical University; www.jichi.ac.jp/saitama-sct/SaitamaHP.files/statmed.html), a graphical user interface for R (version 2.13.0, The R Project for Statistical Computing; http://www.r-project.org) and a modified version of R commander (version 1.8-4).

## Results

After exclusion, a total of 53 patients were included in this study population. In the 53 patients, the original diagnoses were established by CTNB in 2 cases; by EBUS-TBNA in 5 cases; by EBUS-GS in 39 cases; and by surgical biopsy in 7 cases. There were no cases with positive T790M mutation upon initial diagnosis.

For re-biopsy, EBUS–TBNA was performed in 9 patients, whereas EBUS-GS was performed in 44 patients. The technical success rates of the re-biopsy procedures were 100 %. All 9 specimens (100 %) from EBUS-TBNA and 33 specimens (75.0 %) from EBUS-GS were adequate for gene profiling and mutation analysis. The remaining 11 specimens from EBUS-GS procedures were inadequate for mutation analysis owing to the absence of tumor component in the sample (*n* = 6) or insufficient specimen (*n* = 5).

The baseline characteristics of the study population are summarized in Table 1. Initially, EGFR mutation was detected in 53 cases (exon 19 deletion in 30, L858R point mutation in 22, and L861Q point mutation in 1). Among 42 patients that had adequate samples for gene profiling testing for EGFR mutation, 22 (52.4 %) had EGFR mutation and T790M-resistant mutation (Table 2). One initial EGFR-mutated tissue revealed SCLC transformation.

**Table 1:** Patient demographics and clinical information (*N* = 53)

Characteristics	
Age (years)	65 (35–85)
Gender	
Male	19 (35.9)
Female	34 (64.1)
ECOG PS	
0	23 (43.4)
1	27 (50.9)
2	2 (3.8)
3	1 (1.9)
4	0 (0)
Histologic diagnosis	
Adenocarcinoma	52 (98.1)
Adenosquamous cell carcinoma	1 (1.9)
Initial EGFR mutation profile	
Del19	30 (56.6)
L858R	22 (41.5)
L816Q	1 (1.9)
EGFR-TKI treatment before re-biopsy	
Gefitinib	29 (54.8)
Erlotinib	7 (13.2)
Afatinib	0 (0)
Gefitinib + Erlotinib	12 (22.6)
Gefitinib + Afatinib	1 (1.9)
Gefitinib + Erlotinib + Afatinib	4 (7.5)

Data are presented as median (range) or number (%)

*ECOG PS* Eastern Cooperative Group performance status, *EGFR* epidermal growth factor receptor, *EGFR-TKI* EGFR-tyrosine kinase inhibitor

**Table 2:** Biological profiles of cases that underwent re-biopsy by EBUS procedures (*N* = 53)

Initial EGFR mutation profile		EGFR mutation profile on re-biopsy	
Del19	30	Del19 alone	8
		Del19 + T790M	16
		Re-biopsy failure	6
L858R	22	L858R alone	11
		L858R + T790M	6
		Re-biopsy failure	5
L816Q	1	L816Q alone	1

Data are presented as number

*EBUS* endobronchial ultrasound, *EGFR* epidermal growth factor receptor

The adequacy of the re-biopsy specimens for mutation analysis is described in Table 3. The overall detection rate of re-biopsy for malignant cells was 79.2 % (42 of 53); 77.4 % (41 of 53) by cytology and 77.4 % (41 of 53) by histologic examination. The detection rate of re-biopsy by EBUS-TBNA for malignant cells was 100 % (9 of 9), 100 % (9 of 9) by cytology and 88.9 % (8 of 9) by histologic examination. In contrary, the detection rate of re-biopsy by EBUS-GS for malignant cells was 75.0 % (33 of 44); 72.7 % (32 of 44) by cytology and 75.0 % (33 of 44) by histologic examination (Table 3).

**Table 3:** Adequacy of re-biopsy samples for molecular analysis (*N* = 53)

		Detection rate of re-biopsy for malignant cells		
		Cytology	Histology	Overall
ALL	53	41 (77.4)	41 (77.4)	42 (79.2)
EBUS-TBNA	9	9 (100)	8 (88.9)	9 (100)
EBUS-GS	44	32 (72.7)	33 (75.0)	33 (75.0)

Data are presented as number (%)

*EBUS-TBNA* endobronchial ultrasound-guided transbronchial needle aspiration, *EBUS-GS* endobronchial ultrasound with a guide sheath

The factors affecting re-biopsy by EBUS-GS are shown in Table 4. In the multivariate analysis, central parenchymal location and EBUS probe within were the significant predictors of a successful EBUS-GS re-biopsy.

**Table 4:** Factors affecting the yield of re-biopsy by EBUS-GS (*N* = 44)

	Success (*n* = 33)	Failure (*n* = 11)	Univariate	Multivariate	
			*p* value	*p* value	Odds ratio
Age (years)			0.722	–	–
≤70	23 (52.3)	7 (15.9)			
>70	10 (22.7)	4 (9.1)			
Gender			0.282	–	–
Male	13 (29.5)	2 (4.5)			
Female	20 (45.5)	9 (20.5)			
ECOG PS			1.0	–	–
0/1	31 (70.5)	10 (22.7)			
2/3/4	2 (4.5)	1 (2.3)			
Size (mm)			0.139	–	–
≤30	7 (15.9)	5 (11.4)			
>30	26 (59.1)	6 (13.6)			
Location			0.043	0.034	15.7
Central parenchymal	32 (72.7)	8 (18.2)			
Peripheral parenchymal	1 (2.3)	3 (6.8)			
Bronchus sign			0.027	0.239	–
Positive	31 (70.5)	7 (15.9)			
Negative	2 (4.5)	4 (9.1)			
Biopsied pulmonary lesion			0.154	–	–
Primary malignant	30 (68.2)	8 (18.2)			
Metastatic	3 (6.8)	3 (6.8)			
Chest radiograph			0.090	0.080	–
Visible	31 (70.5)	8 (18.2)			
Invisible	2 (4.5)	3 (6.8)			
EBUS			0.016	0.012	10.0
Within	30 (68.2)	6 (13.6)			
Adjacent to/invisible	3 (6.8)	5 (11.4)			

Data are presented as number (%)

*EBUS-GS* endobronchial ultrasound with a guide sheath, *ECOG PS* Eastern Cooperative Group performance status, *EBUS* Endobronchial ultrasound

There were no severe complications after both EBUS-TBNA and EBUS-GS re-biopsy procedures.

## Discussion

Currently, the significance of re-biopsy for mutation analysis of NSCLC has been increasing because of a wider range of therapeutic options. The standard cytotoxic chemotherapy for NSCLC patients has limited therapeutic response [14]. Moreover, after treatment with EGFR-TKIs, kinase inhibition frequently leads to the appearance of drug-resistant mutations within the target kinase itself [15, 16]. Recently, a third generation EGFR-TKI (Osimertinib) has been approved by the US FDA to treat patients with a type of advanced NSCLC that has a specific EGFR mutation, called T790M, and which has become worse after treatment with other EGFR-TKIs. Moreover, Osimertinib has shown clinical effectiveness and tolerability in NSCLC patients with T790M mutation of EGFR [8], underscoring the importance of checking for new mutations after EGFR-TKI therapy in advanced NSCLC patients. There have been several reports about the utility of re-biopsy by CTNB for such purpose.

To our best knowledge, this study was the first to demonstrate the utility of bronchoscopic procedures, especially with EBUS guidance, for mutation analysis of NSCLC after EGFR-TKI therapy. EBUS is a very important procedure to determine and collect samples from target sites in the mediastinal, hilar, and peripheral locations under real-time ultrasound [17, 18]. Adequate sampling of histologic specimens is necessary for the development of new treatment options for cancer, especially chemotherapy and gene-targeted therapy; therefore, further improvements of the histologic sampling yield is essential [19, 20].

EBUS-TBNA is an established minimally invasive procedure for proper staging and diagnosis of lung cancer [10, 21]. In this study, EBUS-TBNA was performed successfully and was able to obtain adequate samples in all cases. EBUS-TBNA is useful not only for proper staging and diagnosis of lung cancer, but also to obtain samples for mutation analysis of NSCLC after EGFR-TKI, as demonstrated in this study. Furthermore, EBUS-TBNA was a safe re-biopsy procedure and had no associated severe complications.

In this study, EBUS-GS was able to obtain samples for mutation analysis of NSCLC after EGFR-TKI. However, when compared with EBUS-TBNA, the detection rate for malignancy was only 75 %. The yield of EBUS-GS for primary diagnosis of PPLs has been reported to be about 70–80 % [22], which is similar to the yield for re-biopsy for mutation analysis in this study. The factors that influence the diagnostic yield of EBUS-GS for PPLs have been reported to be the location of the PPL (central parenchymal or peripheral parenchymal), detected EBUS images (within or adjacent to/invisible), and the presence of a bronchus sign. Although central parenchymal location and detection of EBUS image within were significant factors that predicted a successful yield, the number of peripheral parenchymal cases in this study was small. Further study is needed to confirm the usefulness of EBUS-GS according to the location of the lesion. Although there is need for further technical improvement, EBUS-GS was useful to get samples for mutation analysis of NSCLC after EGFR TKI treatment. In addition, EBUS-GS had no severe complications.

CTNB is another diagnostic procedure for PPLs, with a relatively high rate of both diagnostic accuracy and complications, such as pneumothorax. On the contrary, EBUS-GS was a safe method [23]. However, it is important to note that although EBUS-GS might be considered one of the sampling procedures to safely obtain tissues from PPLs for mutation analysis of NSCLC after EGFR-TKI treatment, the detection rate for malignant cells was significantly lower for peripherally located lesions, as shown in this study. In this regard, EBUS-GS should be performed to get samples for mutation analysis of lesions that are in a central parenchymal location.

Another alternative approach, specifically liquid biopsy, now present as a crucial point in the field [24]. Liquid biopsy has grown in importance because the genetic profile of tumors can affect how well they respond to a certain treatment. A recent paper showed that the concordance between re-biopsy and liquid biopsy, including plasma DNA and circulating tumor cell, was 57–60 % [25]. The usefulness of monitoring T790M status in liquid biopsy was already reported [26]. Although liquid biopsy has the potential to detect new mutations after chemotherapy, several reports have demonstrated some difficulties in detecting tumor-derived mutations in plasma [27]. Therefore, liquid biopsy and re-biopsy may be considered to be complementary methods of mutation analysis.

The limitations of this study were its retrospective and single-institution design. Prospective, multi-center trials are ideal and recommended in the future.

## Conclusions

EBUS procedures for re-biopsy were useful sampling methods for mutation analysis of NSCLC after EGFR-TKI treatment. This could play an important role in the choice of better targeted therapy and for the development of a novel treatment for advanced lung cancer patients.

## Abbreviations

CP-EBUS, convex probe endobronchial ultrasound; CTNB, computed tomography-guided transthoracic needle biopsy; EBUS, endobronchial ultrasound; EBUS-GS, endobronchial ultrasound with a guide sheath; EBUS-TBNA, endobronchial ultrasound guided-transbronchial needle aspiration; EGFR-TKIs, epidermal growth factor receptor-tyrosine kinase inhibitors; NSCLC, non-small cell lung cancer; FDA, Food and Drug Administration; HER2, human epidermal growth factor receptor 2; MET, mesenchymal-to-epithelial transition factor receptor; PPLs, peripheral pulmonary lesions; R-EBUS, radial endobronchial ultrasound
